# Antifungal Combinations against *Candida* Species: From Bench to Bedside

**DOI:** 10.3390/jof8101077

**Published:** 2022-10-13

**Authors:** Simona Fioriti, Lucia Brescini, Francesco Pallotta, Benedetta Canovari, Gianluca Morroni, Francesco Barchiesi

**Affiliations:** 1 Department of Biomedical Sciences and Public Health, Polytechnic University of Marche, 60126 Ancona, Italy; 2 Infectious Disease Clinic, Azienda Ospedaliero Universitaria “Ospedali Riuniti”, 60126 Ancona, Italy; 3 Infectious Diseases Unit, Azienda Ospedaliera Ospedali Riuniti Marche Nord, 61121 Pesaro, Italy

**Keywords:** *Candida* species, antifungals, antifungal susceptibility testing, drug combinations

## Abstract

*Candida* spp. is the major causative agent of fungal infections in hospitalized patients and the fourth most common cause of nosocomial bloodstream infection (BSI). The availability of standardized methods for testing the in vitro activity of antifungals along with the expanding of antifungal armamentarium, the rising of drug-resistance and the persistence of a high mortality rate in systemic candidiasis have led to an increased interest in combination therapy. Therefore, we aimed to review the scientific literature concerning the antifungal combinations against *Candida*. A literature search performed in PubMed yielded 92 studies published from 2000 to 2021: 29 articles referring to in vitro studies, six articles referring to either in vitro and in vivo (i.e., animal models) studies and 57 clinical articles. Pre-clinical studies involved 735 isolates of *Candida* species and 12 unique types of antifungal combination approaches including azoles plus echinocandins (19%), polyenes plus echinocandins (16%), polyenes plus azoles (13%), polyenes plus 5-flucytosine ([5-FC], 13%), azoles plus 5-FC (11%) and other types of combinations (28%). Results varied greatly, often being species-, drug- and methodology-dependent. Some combinatorial regimens exerted a synergistic effect against difficult-to-treat *Candida* species (i.e., azoles plus echinocandins; polyenes plus 5-FC) or they were more effective than monotherapy in prevent or reducing biofilm formation and in speeding the clearance of infected tissues (i.e., polyenes plus echinocandins). In 283 patients with documented *Candida* infections (>90% systemic candidiasis/BSI), an antifungal combination approach could be evaluated. Combinations included: azoles plus echinocandins (36%), 5-FC-combination therapies (24%), polyenes plus azoles (18%), polyenes plus echinocandins (16%) and other types of combination therapy (6%). Case reports describing combination therapies yielded favorable response in most cases, including difficult-to-treat fungal infections (i.e., endocarditis, osteoarticular infections, CNS infections) or difficult-to-treat fungal pathogens. The only randomized trial comparing amphotericin-B deoxycholate (AMB) plus FLU vs. AMB alone for treatment of BSI in nonneutropenic patients showed that the combination trended toward improved success and more-rapid clearance from the bloodstream. In summary, antifungal combinations against *Candida* have produced great interest in the past two decades. To establish whether this approach can become a reliable treatment option, additional in vitro and clinical data are warranted.

## 1. Introduction

*Candida* spp. is the major aetiologic agent of fungal infections in hospitalized patients and the fourth most common cause of nosocomial bloodstream infection [1]. Candidemia, which is associated with high morbidity and mortality, is commonly encountered in hematological, critical care and surgical patients and in even in patients hospitalized in internal medicine wards [2,3]. Although *Candida albicans* remains the most frequent species, the incidence of non-*albicans Candida* infections has been increasing in the last years [4]. *Candida* species other than *C. albicans* (i.e., *C. auris*, *C. krusei*, *C. glabrata* and *C. parapsilosis*) might be more problematic in terms of therapeutic response due to variable susceptibilities of these species to common antifungal agents [5,6].

To date, there are five classes of antifungal drugs used to treat fungal infections: azoles, polyenes, echinocandins, pyrimidines and allylamines [7]. The first two classes, although with unique modes of action, target the same fungal component: membrane ergosterol. While polyenes (i.e., amphotericin B) have potent fungicidal activity against *Candida* spp., azoles (i.e., fluconazole, itraconazole, isavuconazole, posaconazole and voriconazole) are fungistatic. Echinocandins (i.e., anidulafungin, caspofungin and micafungin) target 1,3-β-glucan synthase activity, thus altering cell wall integrity. These drugs are fungicidal against most *Candida* spp. and represent the first choice to treat systemic *Candida* infections [8,9]. Pyrimidines (i.e., 5-flucytosine) and allylamines (i.e., terbinafine) act by inhibiting protein and ergosterol synthesis, respectively. Although allylamines are mainly effective against dermatophytes, anti-*Candida* activity has been also documented [7]. The availability of standardized methods for testing the in vitro activity of antifungals along with the expanding of antifungal armamentarium, the rising of drug-resistance and the persistence of a high mortality rate in systemic candidiasis have led to an increased interest in combination therapy [2,7,10,11].

Therefore, we aimed to review the scientific literature concerning the antifungal combinations against *Candida*. We present the results of combinatorial regimens obtained in vitro, in vivo models and in human infections.

## 2. Materials and Methods

This systematic review was conducted in accordance with the PRISMA guidelines [12]. PubMed was searched with the following string: “pharmacodynamics” and “antifungal combination therapy” and “*Candida*”/“*Diutina”/“Cyberlindnera fabianii*”/“*Debaryomyces hansenii*”/“*Kluyveromyces marxianus*”/“*Meyerozyma guilliermondii*”/“*Pichia*”/“*Wickerhamomyces anomalus*”. A literature search was conducted on 22 November 2021 and included articles published from January 2000 to December 2021. In case of discrepancies in the process of inclusion of papers/data extraction, a consensus was reached through discussion or involvement of the authors. Additional cases were sought from the reference list of included papers. The inclusion criteria were antifungal combinations for *Candida* species. The exclusion criteria were papers not referring to antifungal combinations (i.e., combination of a given antifungal with a chemical compound other than antifungal), papers with unspecified combination therapy, literature reviews, papers in languages other than English, papers considered off-topics and papers without fungal identification. Data from the included papers were entered in a database, created with Excel, which encompassed the genus/species/number of *Candida* isolates tested, the type of drug combination, the method utilized for testing and the results of the interaction. In the case of clinical reports, demographic data (when available) and outcome of the combination therapy were reported.

## 3. Results and Discussion

**Figure 1:**
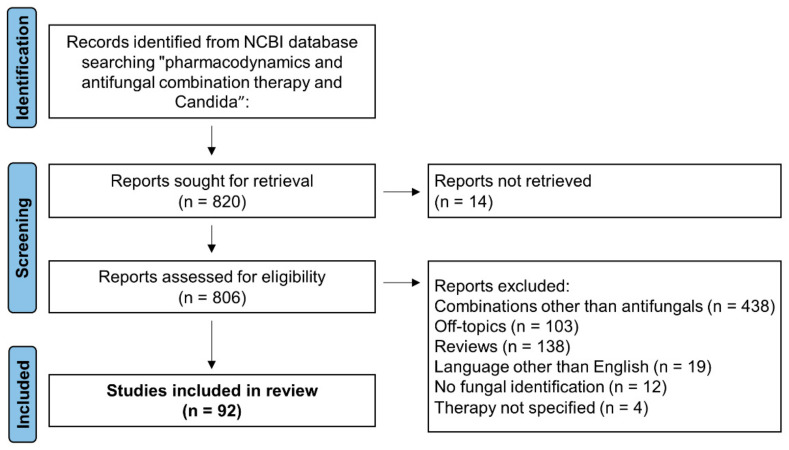
Flowchart of article selection process of this review.

A total of 820 articles were initially identified (Figure 1). Upon removal of reports not retrieved (n = 14), a total of 806 papers were assessed for eligibility. Among them, we excluded 714 reports for the following reasons: combinations other than antifungals (n = 438), reviews (n = 138), off-topics (n = 103), languages other than English (n = 19), no fungal identification (n = 12) and unspecified combination therapy (n = 4). Therefore, a total of 92 studies were included in this review [13,14,15,16,17,18,19,20,21,22,23,24,25,26,27,28,29,30,31,32,33,34,35,36,37,38,39,40,41,42,43,44,45,46,47,48,49,50,51,52,53,54,55,56,57,58,59,60,61,62,63,64,65,66,67,68,69,70,71,72,73,74,75,76,77,78,79,80,81,82,83,84,85,86,87,88,89,90,91,92,93,94,95,96,97,98,99,100,101,102,103,104,105]: 29 articles referring only to in vitro studies, six articles referring to either in vitro and in vivo (i.e., animal models) studies and 57 articles referring only to in vivo studies (Table 1, Table 2 and Table S1). Among the latter group of papers, there were 55 articles referring to clinical cases (Table 3 and Table S2).

### 3.1. In Vitro Studies and Experimental Models of Infection

**Figure 2:**
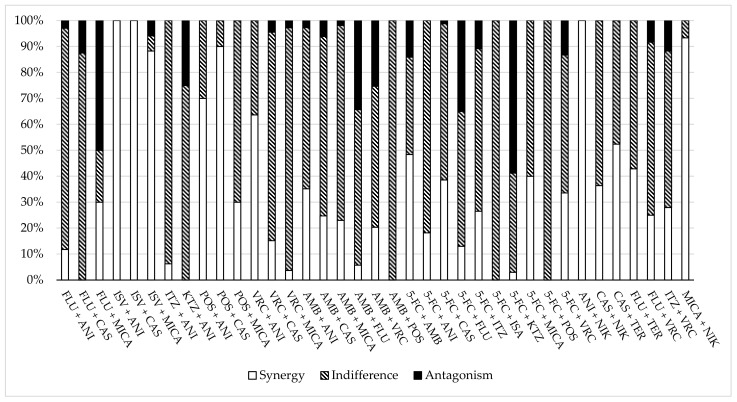
Percentage of synergy, indifference and antagonism of the antifungal combinations in in vitro experiments.

The results of in vitro antifungal combinations and of experimental models of infection are reported in Table 1, Table 2 and Table S1 and Figure 2. A total of 735 isolates of *Candida* species were tested. There were 257 isolates of *Candida albicans* (23 studies), 137 of *Candida glabrata* (14 studies), 111 of *Candida parapsilosis* (11 studies), 80 of *Candida dubliniensis* (3 studies), 56 of *Candida krusei* (9 studies), 35 of *Candida tropicalis* (8 studies), 28 of *Candida kefyr* (2 studies), 21 of *Candida auris* (2 studies) and 10 of *Candida lusitaniae* (1 study). A total of 12 unique types of antifungal combination approaches were experimented: azoles plus echinocandins (19%), polyenes plus echinocandins (16%), polyenes plus azoles (13%), polyenes plus 5-FC (13%), azoles plus 5-FC (11%) and other types of combinations (28%). The latter group of combinations included: echinocandins plus either 5-FC or the inhibitor chitin synthase, nykkomycin, double azoles, unique triple combinations, terbinafine combined with either azoles or echinocandins and double echinocandins. Checkerboard titration methodologies, alone or combined with killing experiments, were the most common procedures for testing the in vitro combination. In most cases, the type of interaction was defined by the Fractional Inhibitory Concentration Index (FICI) (Table S1).

#### 3.1.1. Azoles plus Echinocandins

The interactions between these molecules ranged from a synergistic to an antagonistic effect, with the most common effect being indifference. One study investigated the effects of VRC in combination with MICA against a large collection of *Candida* isolates and found 97% indifference regardless the species tested [13]. Another study investigated the interactions of several azoles (FLU, ITZ and ketoconazole [KTZ]) with ANI against four isolates each of *C. albicans*, *C. glabrata*, *C. parapsilosis*, *C. tropicalis* and two isolates of *C. krusei* and found that additive activity or indifference was observed in 85 of 90 interactions [14]. Synergy with ITZ was observed for one isolate of *C. glabrata*, and antagonism with KTZ was noted in four isolates of *C. tropicalis*. Siopi et al., compared the classic checkerboard method with a gradient assay and found that combination of VRC and CAS yielded variable results which were species-dependent with antagonism occurring in *C. parapsilosis*, *C. krusei* and *C. tropicalis*, additivism in *C. albicans* and *C. kefyr* and synergism in *C. glabrata* [15]. The last triazole introduced in clinical practice, ISV, was tested in combination with all three echinocandins [16,17]. One study aimed to determine the in vitro interactions of ISV with echinocandins against MDR yeast *C. auris*. Interactions were determined using a checkerboard method, and absorbance data were analyzed by FICI, the Greco universal response surface approach and the Bliss interaction model. All models showed that the combinations were more effective than monotherapy regimens while time-kill experiments revealed that once synergy was achieved, combinations of higher drug concentrations did not improve antifungal activity (i.e., fungicidal activity) [16]. Another study used Bliss interaction analysis and time-kill assays to examine the in vitro interactions of ISV and MICA against five species of *Candida* and found that the combination resulted in synergistic interactions against *C. albicans*, *C. parapsilosis* and *C. krusei* with the highest synergy occurring against *C. albicans*. Additionally, time-kill experiment showed that the combination demonstrated concentration-dependent synergy against *C. albicans* and *C. parapsilosis* [17]. Even experimental models of infections due to *C. albicans* showed variable results. One study evaluated combinations of FLU with CAS in murine candidemia and did not show any benefit of combined therapy over individual antifungal drugs in terms of number of CFU/kidney tissue [18]. Another study investigated the combination of POS plus CAS in a mouse model of systemic infection and found a significantly longer survival of mice treated with the combination with respect to those treated with monotherapy [19].

Overall, these data showed that azoles combined with echinocandins yield variable results which are species-, drug- and methodology-dependent. It is interesting to note that this combination therapy often yielded synergistic interaction against difficult-to-treat *Candida* species such as *C. auris*, *C. glabrata* and *C. krusei*. Further studies are necessary to corroborate these findings.

#### 3.1.2. Polyenes plus Echinocandins

AMB combined with CAS was the most frequent experimented combination. Several studies investigated this approach against clinical isolates of *C. glabrata* [20,21,22]. An early study examined this interaction in vitro and in the experimental model of murine infection. Although MICs of both drugs given in combination were generally lower than those observed when the drugs were tested alone, indifference was the only type of interaction observed by a checkerboard method. Similarly, an indifferent effect was observed in time-kill studies. On the other hand, when both drugs were administered at dosages of 1 mg/kg/day, the combination regimen was the only therapeutic approach yielding organ sterilization in a neutropenic mice model [20]. Similarly, Olson et al. used immunosuppressed mice infected with *C. glabrata* to investigate the efficacy of L-AMB alone or in combination with CAS or MICA and found that complete clearance of infection could be achieved only when drugs were given in combination or if L-AMB was given sequentially with CAS [23]. More recent in vitro investigations utilizing many *C. glabrata* clinical isolates confirmed that the polyene combined with CAS yielded an indifferent interaction in many cases (66–70%) while synergy was seen in about 20% of cases. Antagonism was rarely found [21,22]. Similar results were obtained when the polyene was combined with ANI while the combination with MICA yielded a higher frequency of antagonism (50%) [21]. Again, when AMB was combined with echinocandins against clinical isolates of *C. albicans,* indifference was the interaction more often observed [24,25,26]. The only exception was represented by one study investigating the effect of ANI with the polyene vs. three isolates each of *C. albicans*, *C. tropicalis* and *C. parapsilosis* grown either as planktonic or sessile cells obtained from biofilms formed on polyurethane central venous catheters [27]. Both ANI and AMB exerted excellent activity against both forms of cells, which was significantly augmented upon the combination. Another study employing in vitro and experimental mouse models analyzed the effect of AMB combined with CAS against three isolates of *C. parapsilosis*. The in vitro results were methodology-dependent with the disk diffusion assay showing halo diameters produced by combinations significantly greater than those produced by each drug alone. Similarly, low doses of CAS (i.e., 0.25 to 1 mg/kg/day) combined with AMB at 1 mg/kg/day were significantly more effective than each single drug at reducing the colony counts in kidneys. Higher doses of the echinocandin combined with the polyene did not show any advantage over CAS alone [28].

Overall, these findings show some potential of this combination. Although in studies employing classical in vitro methodologies such as checkerboard or killing curves a synergistic interaction was rarely seen, the addition of an echinocandin to the polyene might be advantageous in speeding the clearance of infected tissues or to reduce or prevent the risk of biofilm formation.

#### 3.1.3. Polyenes plus Azoles

Although polyenes and azoles target the same fungal membrane component, ergosterol, they do so by unique mechanisms of action. Therefore, a potential for increasing the antifungal activity upon combination can occur. Indeed, literature data showed a great interest for this combination as well. Early studies experimented the combination of AMB with FLU vs. a limited number of *Candida* isolates belonging to four species and found that antagonism was frequent, occurring in 50% and 100% of the cases when tests were done with the checkerboard and the gradient concentration strips diffusion methodologies, respectively [29,30]. To investigate the complex interaction between AMB and FLU, one early study used in vitro time-kill experiments and a rabbit model of *C. albicans* endocarditis and pyelonephritis and found that preexposure of *C. albicans* to FLU reduces fungal susceptibility to AMB. In vivo, AMB monotherapy and treatment with AMB for 24 h followed by AMB plus FLU rapidly sterilized kidneys and cardiac vegetations. AMB plus FLU or FLU followed by AMB treatments were slower in clearing fungi from infected tissues [30]. A later study addressing the in vitro effects of AMB plus FLU against *C. parapsilosis* (n = 60) confirmed that antagonism was not a rare event occurring in 33% of the cases, while indifference/additivism characterized most of the cases [31]. The addition of VRC to AMB produced variable results which were either species- or methodology-dependent [15,22,31]. One study investigated this interaction against multiple clinical isolates belonging to six *Candida* species by two methods: the classical checkerboard and the gradient concentration strips diffusion [32]. While the latter method yielded antagonism in almost all cases (95%), the checkerboard produced synergistic results in 33% of cases and indifference was seen in the remaining cases. One study focused this combination vs. many isolates belonging to *C. glabrata* and found that, although indifference was the most frequent observed phenomenon, synergy did not occur rarely being found in 31% of the cases [22]. Finally, when AMB was either combined with ISV or POS, a reciprocal drug potentiation was seen rarely while indifference (45–100%) or even antagonism (22–39%) were frequently observed.

Overall, these data seem to indicate that this combination is not useful, indeed it could cause a reduction of the antifungal effect in some circumstances. It remains to be verified whether in the case of yeasts with innate reduced sensitivity to azoles (e.g., *C. glabrata*) the addition of polyene can lead to an enhancement of the antifungal effect.

#### 3.1.4. 5-FC Combination Therapies

5-FC has a unique mechanism of action which theoretically makes it identifiable as an ideal partner drug. In most in vitro studies, this drug has been utilized in combination with AMB, followed by the combinations with azoles and echinocandins. Several investigations have been performed by using difficult-to-treat *Candida* species. A recent study utilized two-drug combinations against multidrug-resistant *C. auris* (n = 15) by measuring 100% inhibition as endpoints and found that 5-FC at 1.0 mg/L potentiated the most combinations [33]. Specifically, for nine *C. auris* isolates resistant to AMB (MIC  ≥  2.0 mg/L), AMB-5-FC (0.25/1.0 mg/L) yielded 100% inhibition, six isolates resistant to three echinocandins (ANI/MICA MIC  ≥  4.0 mg/L, CAS MIC  ≥  2.0 mg/L) were 100% inhibited by ANI-5-FC and CAS-5-FC (0.0078/1 mg/L) and MICA-5-FC (0.12/1 mg/L) and 13 isolates with a high VRC MIC (>2 mg/L) were 100% inhibited by the VRC-5-FC (0.015/1 mg/L) [33]. An early study evaluated combinations of antifungals in a checkerboard assay against two groups of *C. glabrata* clinical isolates (n = 68): one containing FLU-susceptible (FS) and another containing FLU-resistant isolates (FR). The most synergistic combination observed was 5-FC plus AMB (synergistic for 62% of FS and 76% FR isolates). The most antagonistic combination observed was 5-FC plus KTZ (FS 62% and FR 56%). Most combinations evidenced indifferent interactions [21]. Another study focused the interactions between several antifungal agents, including 5-FC, against a large collection of *C. parapsilosis* (n = 60) clinical isolates consisting of echinocandin-resistant and -susceptible strains. Synergy was rarely seen while indifference/antagonism, which were the most common types of interaction, characterized the following percentages of cases: 5-FC-AMB 50/22%, 5-FC-FLU 58/27% and 5-FC-VRC 53/22% [31]. Several reports investigated the interactions between 5-FC and other drugs using clinical isolates belonging to various *Candida* species. One study experimented the efficacy of 5-FC in combination with AMB and FLU against nine isolates each *C. albicans*, *C. glabrata* and *C. krusei* using a broth microdilution checkerboard method and measuring growth by estimation from the response surface approach. The 5-FC-AMB combination approach yielded synergy/antagonism in 44/56%, 33/67% and 77/22% of isolates of *C. albicans*, *C. glabrata* and *C. krusei*, respectively. The 5-FC-FLU combination approach yielded synergy/antagonism in 50/50%, 0/100% and 11/89% of isolates of *C. albicans*, *C. glabrata* and *C. krusei*, respectively [34]. Another study investigated the same combinatorial regimens against three isolate of *C. albicans* and one isolate each of *C. glabrata*, *C. krusei* and *C. tropicalis* by checkerboard, killing experiments and E-test methods. Indifference was the most common type of interaction regardless of drug combination where *Candida* species and methodology was employed [29]. 

Overall, these in vitro data indicate that 5-FC combined with AMB might be advantageous in some cases, having shown a reciprocal potentiation in difficult-to-treat *Candida* species (i.e., *C. auris*, *C. glabrata* and *C. krusei*). The addition of 5-FC to drugs belonging to other classes does not seem to have a beneficial effect. However, further studies are needed to corroborate these findings. 

#### 3.1.5. Other Combinations

Nikkomycin Z (NIK) acts as inhibitor of chitin synthase. It has been postulated that a chitin synthase inhibitor combined with various antifungal agents can be significantly synergistic against a range of medically important fungi [35]. Recently, this drug has been experimented in combination with several echinocandins. One study evaluated the in vitro and in vivo effects of NIK combined with ANI or MICA against two *C. albicans* isolates and their lab-derived echinocandin-resistant *fks* mutants. Synergism was observed for all strains. Combination treatment with NIK and either echinocandin significantly improved the survival rate of mice infected with the *fks* mutants compared with that of mice treated with NIK or echinocandin monotherapy, suggesting the therapeutic potential of this combination in managing echinocandin resistance [36]. Another study examined the interactions between two echinocandins, CAS and MICA with NIK, against *C. albicans* and *C. parapsilosis* biofilms using the XTT-based checkerboard microdilution method, and the nature of interactions was assessed by calculating fractional inhibitory concentration indices and using the Bliss independence model. Both echinocandins combined with NIK caused an extended cell death and the structure of the biofilm was sparse compared to the control, especially for *C. albicans,* suggesting a reciprocal drug potentiation [37]. The allylamine derivative, TER, was studied in combination with either echinocandins or azoles [38,39,40]. One early study investigated the activity of CSP combined with TRB against *C. dubliniensis*, *C. kefyr* and azole-resistant *C. albicans* by checkerboard analysis. The combination of CAS with TER resulted in positive interactions against *C. albicans* and *C. kefyr* but not against *C. dubliniensis*. Additionally, true synergism was observed only against TRB-resistant strains which became susceptible to this drug in the presence of CAS [38]. Another study analyzed antifungal activities of FLU and TER alone and in combination against *C. albicans* and found synergy in 50% of the cases, while antagonism was never observed. Moreover, the combination significantly decreased the expression levels of the *ERG1*, *ERG3* and *ERG11* genes, suggesting that FLU plus TER could destroy the cell membrane through the inhibition of all three key enzymes in the ergosterol biosynthesis of *C. albicans* [40]. 

Triple combination therapies were also conducted in in vitro models mimicking difficult-to-treat infections such as endocarditis. One study investigated the effects of combinations of 5-FC, MICA and VRC against *Candida*-infected human platelet-fibrin clots, used as simulated endocardial vegetations, and found that the triple therapy was no better than single or dual agents against any of the four *Candida* species tested (i.e., *C. albicans*, *C. glabrata*, *C. parapsilosis* and *C. tropicalis)* [41]. Another study analyzed the in vitro effects of 5-FC, L-AMB and MICA combinations against two *Candida albicans* strains that simulated 24-h-old endocardial vegetations. The combination was superior to all other treatments for one strain but no different from the dual combination of L-AMB-MICA for the other strain [42].

### 3.2. Clinical Cases

The results of antifungal combinations in humans are reported in Table 3 and Table S2. A total of 286 patients were described. There were 52 papers describing 139 single cases, one open-label, noncomparative, clinical trial involving a total of 29 patients and one randomized clinical trial involving a total of 118 patients [43,44,45,46,47,54,56,57,58,59,60,61,62,63,64,65,66,67,68,69,70,71,72,73,74,75,76,77,78,79,80,81,82,83,84,85,86,87,88,89,90,91,92,93,94,95,96,97,98,99,100,101,102,103,104,105]. Either pediatric or adult patients were represented. In most of the patients (93%), combination therapy was used for a systemic infection. Combination therapies described in the case reports included: azoles plus echinocandins (36%), 5-FC-combination therapies (24%), polyenes plus azoles (18%), polyenes plus echinocandins (16%) and other types of combination therapy (6%). Case reports describing combination therapies yielded favorable responses in most cases, including difficult-to-treat fungal infections (i.e., endocarditis, osteoarticular infections, CNS infections) or difficult-to-treat fungal pathogens (i.e., infections due to *C. glabrata* or *C. krusei*). A retrospective study investigated the effects of the addition of CAS to conventional antifungals for the treatment of refractory candidemia (persisting infection despite 6 to 30 days of conventional antifungal therapy) in 12 preterm infants. The combination yielded sterilization of blood cultures in 11 infants at the median time of 3 days, showing that this approach might be efficacious in this infection [43]. One international, open label, noncomparative, clinical trial investigated the efficacy of MICA alone and in combination with other antifungal drugs for treatment of newly diagnosed and refractory candidemia [44]. The outcome of 29 patients treated with a combination therapy (any antifungal drug plus MICA) was compared with that of 25 patients treated with MICA alone. Overall success was obtained in 79.3% vs. 76% of patients treated with combination and monotherapy, respectively. 

A prospective study described 30 patients with *Candida* endocarditis. Most of the patients (80%) were given combination therapy, which was associated with surgery in 43% of the cases. The following drugs, variously combined with each other, were utilized at decreasing frequencies: CAS (77%), 5-FC (70%), FLU (60%), AMB (47%) and VRC (30%). No therapeutic option gave a survival benefit [45]. One study evaluated AMB combined with 5-FC in fungal peritonitis (FP) due to *Candida* species in 13 patients under peritoneal dialysis with deferred catheter replacement (26 ± 7.7 days upon initial treatment) and compared their outcome with 14 historic controls treated with AMB, FLU or a combination of the two, and most of whose catheters were removed before day 10 of presentation. It was found that the study group appeared to have a significantly lower technique failure rate, similar mortality and similar of length hospitalization [46]. 

Rex et al. conducted a randomized, blinded and multicenter trial to compare FLU plus placebo with FLU plus AMB deoxycholate for treatment of candidemia in nonneutropenic adult patients. A total of 219 patients were included in the modified intent-to-treat analysis. The overall success rates were 56% (60 of 107 patients) of FLU plus placebo vs. 69% (77 of 112 patients; *p* = 0.043) of FLU plus AMB while the bloodstream infection failed to clear in 17% and 6% of subjects, respectively (*p* = 0.02). The authors conclude that in nonneutropenic subjects, the combination of FLU plus AMB was not antagonistic compared with FLU alone; rather, the combination trended toward improved success and more-rapid clearance from the bloodstream [47].

Overall, these data indicate that antifungal combination therapy has been used in a wide variety of disparate clinical conditions making the results difficult to interpret. Since in most cases, a favorable outcome is highlighted, it is possible to hypothesize that there is an under-representation of those cases with negative results. In some clinical circumstances (e.g., uncomplicated bloodstream infections in immunocompetent patients), even combinatorial regimens not strongly supported by in vitro results (e.g., azoles plus polyenes) may have some benefit. On the contrary, in more complex clinical situations (e.g., endocarditis), even the combination of fungicidal drugs with excellent in vitro performance (e.g., echinocandins plus polyenes) may not be adequate to determine a positive outcome of the infection. Clearly, additional randomized clinical trials are needed to further elucidate this strategy.

## 4. Conclusions

The international guidelines for the management of candidiasis generally do not contemplate a combination therapy except in some clinical circumstances such as CNS infections and endocarditis in which 5-FC can be added to AMB [8]. In the last 20 years, however, there has been considerable scientific interest in combination therapy in *Candida* infections, as demonstrated by the numerous scientific reports published on this topic. While it is difficult to draw firm conclusions that might be helpful in making a treatment decision, some considerations need to be made. 

One of the main limitations of our review is related to the research string using “pharmacodynamics”, “antifungal” and “Candida”, which may have led to an underestimation of the articles specific of this issue.

In vitro results vary greatly, being often species-, drug- and methodology-dependent. It must be noted that, in most in vitro experiments, the reproducibility of the procedure was not taken into consideration. In this regard, it would be very useful to carry out multicenter studies to analyze the reproducibility of these results. Interestingly, some combinatorial regimens exerted a synergistic effect against difficult-to-treat *Candida* species (i.e., *C. glabrata*, *C. krusei*, *C. auris*) or they were more effective than monotherapy in prevent or reducing biofilm formation. Both these issues need to be further investigated with a larger number of isolates and multiple methodologies. In vivo results (either in experimental models or in humans) often showed some advantages of the combination vs. monotherapy in clearing the infection even when molecules targeting the same fungal component are used. Although most case reported the combination turning out to be effective, yielding a “full recovery”, the lack of any control makes the strength of conclusions weaker.

Following the increasing identification of antifungal resistant isolates and the challenge in the management of these infections, a combination regimen could represent an advantage in these clinical circumstances. Several resistance mechanisms in the last decade have been detected and in the next years they could become a worrying problem [106]. Additional studies on antifungal combinations and their efficacy on resistance isolates could help in facing these complicated infections. 

Alongside the antifungals combinations, studies on new molecules and alternative therapies could be critical to increase the efficacy of the current treatment of *Candida* infections. Some new antifungal drugs are currently in development and included both molecules with old targets, such as ibrexafungerp or rezafungin, which represent an evolution of echinocandins, and novel compounds with new mechanisms of action, such as otesaconazole and fosmanogepix (targeting the lanosterol demethylase) [106]. 

Furthermore, novel natural compounds such as peptides, lipopeptides or retinoids also showed efficacy in the inhibition of *Candida* growth [107,108,109]. Although the results are still preliminary, the studies of these new molecules could enhance the knowledge of antifungal targets and usher in the development of new therapeutic options against fungal infections. 

In summary, antifungal combinations against *Candida* have produced great interest in the past two decades. To establish whether this approach can become a reliable treatment option, additional in vitro and clinical data are warranted.

## Figures and Tables

**Table 1:** Cumulative number of in vitro combinations results reported in the studies analyzed.

	*C. alb*			*C. par*			*C. gla*			*C. tro*			*C. aur*			*C. kru*			*C. dub*			*C. lus*			*C. kef*			All		
	S	I	A	S	I	A	S	I	A	S	I	A	S	I	A	S	I	A	S	I	A	S	I	A	S	I	A	S	I	A
Azoles + Echinocandins																														
FLU + ANI	-	6	-	-	4	-	4	9	1	-	6	-	-	-	-	-	4	-	-	-	-	-	-	-	-	-	-	4	29	1
FLU + CAS	-	2	-	-	-	-	-	8	2	-	2	-	-	-	-	-	2	-	-	-	-	-	-	-	-	-	-	0	14	2
FLU + MICA	-	-	-	-	-	-	3	2	5	-	-	-	-	-	-	-	-	-	-	-	-	-	-	-	-	-	-	3	2	5
ISV + ANI	-	-	-	-	-	-	-	-	-	-	-	-	12	-	-	-	-	-	-	-	-	-	-	-	-	-	-	12	0	0
ISV + CAS	-	-	-	-	-	-	-	-	-	-	-	-	12	-	-	-	-	-	-	-	-	-	-	-	-	-	-	12	0	0
ISV + MICA	1	-	-	1	-	-	-	-	1	-	1	-	12	-	-	1	-	-	-	-	-	-	-	-	-	-	-	15	1	1
ITZ + ANI	-	4	-	-	2	-	1	3	-	-	4	-	-	-	-	-	2	-	-	-	-	-	-	-	-	-	-	1	15	0
KTZ + ANI	-	4	-	-	2	-	-	4	-	-	-	4	-	-	-	-	2	-	-	-	-	-	-	-	-	-	-	0	12	4
POS + ANI	-	-	-	-	-	-	7	3	-	-	-	-	-	-	-	-	-	-	-	-	-	-	-	-	-	-	-	7	3	0
POS + CAS	10	-	-	-	-	-	8	2	-	-	-	-	-	-	-	-	-	-	-	-	-	-	-	-	-	-	-	18	2	0
POS + MICA	-	-	-	-	-	-	3	7	-	-	-	-	-	-	-	-	-	-	-	-	-	-	-	-	-	-	-	3	7	0
VRC + ANI	-	-	-	-	1	-	7	3	-	-	-	-	-	-	-	-	-	-	-	-	-	-	-	-	-	-	-	7	4	0
VRC + CAS	-	30	-	-	1	-	17	59	5	-	-	-	-	-	-	-	-	-	-	-	-	-	-	-	-	-	-	17	90	5
VRC + MICA	1	54	-	-	13	-	3	16	3	-	-	-	-	-	-	-	-	-	-	19	-	-	-	-	-	-	-	4	102	3
Polyenes + Echinocandins																														
AMB + ANI	3	4	-	3	4	-	4	9	1	3	4	-	-	-	-	-	2	-	-	-	-	-	-	-	-	-	-	13	23	1
AMB + CAS	4	7	-	-	6	-	20	54	6	-	-	-	-	-	-	-	-	-	-	-	-	-	-	-	-	-	-	24	67	6
AMB + MICA	4	33	-	7	10	-	3	21	3	7	8	-	-	-	-	9	26	-	7	13	-	-	10	-	-	-	-	37	121	3
Polyenes + Azoles																														
AMB + FLU	-	3	4	3	37	20	1	-	-	-	1	-	-	-	-	-	1	-	-	-	-	-	-	-	-	-	-	4	42	24
AMB + VRC	-	-	-	-	35	25	28	40	10	-	-	-	-	-	-	-	-	-	-	-	-	-	-	-	-	-	-	28	75	35
AMB + POS	-	10	-	-	-	-	-	-	-	-	-	-	-	-	-	-	-	-	-	-	-	-	-	-	-	-	-	0	10	0
5-FC combination																														
5-FC + AMB	5	4	5	12	35	13	55	23	6	1	-	-	16	6	2	-	1	-	-	-	-	-	-	-	-	-	-	89	69	26
5-FC + ANI	-	4	-	-	4	-	-	4	-	-	4	-	6	9	-	-	2	-	-	-	-	-	-	-	-	-	-	6	27	0
5-FC + CAS	-	-	-	-	-	-	26	41	1	-	-	-	6	9	-	-	-	-	-	-	-	-	-	-	-	-	-	32	50	1
5-FC + FLU	6	1	5	4	40	16	1	1	14	1	-	-	-	-	-	1	10	-	-	-	-	-	-	-	-	-	-	13	52	35
5-FC + ITZ	-	-	-	-	-	-	22	37	9	-	-	-	-	15	-	-	-	-	-	-	-	-	-	-	-	-	-	22	52	9
5-FC + ISA	-	-	-	-	-	-	-	-	-	-	-	-	-	15	-	-	-	-	-	-	-	-	-	-	-	-	-	0	15	0
5-FC + KTZ	-	-	-	-	-	-	2	26	40	-	-	-	-	-	-	-	-	-	-	-	-	-	-	-	-	-	-	2	26	40
5-FC + MICA	-	-	-	-	-	-	-	-	-	-	-	-	6	9	-	-	-	-	-	-	-	-	-	-	-	-	-	6	9	0
5-FC + POS	-	-	-	-	-	-	-	-	-	-	-	-	-	15	-	-	-	-	-	-	-	-	-	-	-	-	-	0	15	0
5-FC + VRC	-	-	-	10	37	13	25	37	6	-	-	-	13	2	-	-	-	-	-	-	-	-	-	-	-	-	-	48	76	19
Other combinations																														
ANI + NIK	4	-	-	-	-	-	-	-	-	-	-	-	-	-	-	-	-	-	-	-	-	-	-	-	-	-	-	4	0	0
CAS + NIK	4	2	-	-	5	-	-	-	-	-	-	-	-	-	-	-	-	-	-	-	-	-	-	-	-	-	-	4	7	0
CAS + TER	40	19	-	-	-	-	-	-	-	-	-	-	-	-	-	-	-	-	-	41	-	-	-	-	26	-	-	66	60	0
FLU + TER	3	4	-	-	-	-	-	-	-	-	-	-	-	-	-	-	-	-	-	-	-	-	-	-	-	-	-	3	4	0
FLU + VRC	-	-	-	15	40	5	-	-	-	-	-	-	-	-	-	-	-	-	-	-	-	-	-	-	-	-	-	15	40	5
ITZ + VRC	-	-	-	-	-	-	19	41	8	-	-	-	-	-	-	-	-	-	-	-	-	-	-	-	-	-	-	19	41	8
MICA + NIK	10	-	-	4	1	-	-	-	-	-	-	-	-	-	-	-	-	-	-	-	-	-	-	-	-	-	-	14	1	0

The table included results regardless the methods used to assess the interaction between antifungals. Reports with specific endpoints or using more than two antifungals in combinations were not included. Interactions for checkerboards were defined as synergistic (S) if the FIC index (FICI) was ≤0.5, indifferent (I) if 0.5 ≤ FICI ≤ 4.0, and antagonistic (A) if FICI was >4.0. Interactions for time kill assays were defined as synergistic (S) if combination resulted in a CFU reduction >2Log compared to most active drug, indifferent (I) if the combination yielded a CFU number ≤±2Log compared to the most active drug, and antagonistic (A) if the CFU number of the combinations was higher than 2Log compared to the most active drug. Abbreviations: *C. alb*, *C. albicans*; *C. par*, *C. parapsilosis*; *C. gla*, *C. glabrata*; *C. tro*, *C. tropicalis*; *C. aur*, *C. auris*; *C. kru*, *C. krusei*; *C. dub*, *C. dubliniensis*; *C. lus*, *C. lusitaniae*, *C. kef*, *C. kefyr*; ISV, isavuconazole; ANI, anidulafungin; CAS, caspofungin; MICA, micafungin; AMB, amphotericin; 5-FC, 5-flucytosine; POS, posaconazole; ITZ, itraconazole; VRC, voriconazole; NIK, nikkomicin Z; FLU, fluconazole; TER, terbinafine; KTZ, ketoconazole.

**Table 2:** Reports of antifungals combinations in experimental animal model of infections.

Reference	Isolates and Species	Combinations	Methods	Results
**Kalkanci et al., 2018 [58]**	12 corneas were inoculated with *C. albicans*	VRC + AMB	Corneal Infection Rabbit model	Two Log reduction in colony numbers compared to single treatment
**Alvarez et al., 2017 [48]**	C. albicans *	AMB + 5-FC	Systemic Infection Neutropenic Mouse model	No differences compared to monotherapy
**Chen et al., 2013 [19]**	Three C. albicans *	POS + CAS	Systemic Infection Mouse model	SYN in 1 isolate, NO SYN in drug resistant isolates
**Olson et al., 2005 [23]**	*C. glabrata*	AMB + CAS or AMB + MICA	Systemic Infection Neutropenic Mouse model	Improved activity of combination therapy
**Barchiesi et al., 2005 [20]**	C. glabrata *	CAS + AMB	Systemic Infection Neutropenic Mouse model	>100 fold CFU difference
**Graybill et al., 2003 [18]**	*C. albicans*	CAS + FLU	Systemic Infection Mouse model	No differences compared to monotherapy
**Hossain et al., 2003 [26]**	C. albicans *	CAS + AMB	Systemic Infection Mouse model	CAS + AMB prolonged survival compared with untreated control. Treatment of MICA with AMB + CAS, even at low dosage also tended to prolong survival
**Louie et al., 2001 [30]**	*C. albicans*	FLU + AMB	Rabbit model of endocarditis and pyelonephritis	No differences compared to monotherapy

* The study used isolates resistant to at least one antifungal drug. Abbreviations: CAS, caspofungin; MICA, micafungin; AMB, amphotericin; 5-FC, 5-flucytosine; POS, posaconazole; VRC, voriconazole; FLU, fluconazole.

**Table 3:** Number and percentage of clinical success or failure of the different antifungals’ combinations used in case reports and clinical trials reported in the study.

Combinations	Number of Cases	Success n (%)	Failure n (%)
AMB + FLU	142	100 (70.4%)	42 (29.6%)
AMB + 5-FC	24	15 (62.5%)	9 (37.5%)
AMB + CAS	15	8 (53.3%)	7 (46.7%)
CAS + 5-FC	13	4 (30.8%)	9 (69.2%)
AMB + CAS + FLU	11	5 (45.5%)	6 (54.5%)
CAS + VRC	9	7 (77.8%)	2 (22.2%)
FLU + 5-FC	8	6 (75.0%)	2 (25.0%)
CAS + FLU	6	4 (66.7%)	2 (33.3%)
FLU + MICA	4	3 (75.0%)	1 (25.0%)
AMB + CAS + VRC	2	2 (100%)	-
AMB + 5-FC + FLU	1	1 (100%)	-
AMB + 5-FC + VRC	1	1 (100%)	-
AMB + ANI	1	1 (100%)	-
AMB + FLU + 5-FC + CAS	1	1 (100%)	-
AMB + FLU + MICA	1	1 (100%)	-
AMB + KTZ	1	1 (100%)	-
AMB + KTZ	1	1 (100%)	-
AMB + VRC	1	1 (100%)	-
CAS + FLU + POS	1	1 (100%)	-
CAS + ITZ	1	-	1 (100%)
FLU + VRC	1	-	1 (100%)
ITZ + EFI	1	-	1 (100%)
MICA + VRC	1	1 (100%)	-

Abbreviations: CAS, caspofungin; AMB, amphotericin B; VRC, voriconazole; FLU, fluconazole; ITZ, itraconazole; EFI, eficonazole; MICA, micafungin; 5-FC, 5-flucytosine; ANI, anidulafungin; KTZ, ketoconazole. Some studies were omitted due to the impossibility to match therapies and outcome. In the case of multiple combination therapy, only the last combination used were used in this table. In some cases, success and failure were reported as microbiological and not clinical.

## Data Availability

All data used in this study were obtained from PubMed (https://pubmed.ncbi.nlm.nih.gov/, accessed on 22 November 2021).

## Supplementary Materials

The following supporting information can be downloaded at: https://www.mdpi.com/article/10.3390/jof8101077/s1, Table S1. In vitro papers using antifungal combinations; Table S2. Case reports and clinical trials using antifungal combinations.
