# Supplementary material for: Antifungal Combinations against Candida Species: From Bench to Bedside
Source: J Fungi (Basel). 2022 Oct 13;8(10):1077. doi: 10.3390/jof8101077 (PMC9605143; doi:10.3390/jof8101077)
Supplement: Supplementary file 1 [file jof-08-01077-s001.zip › jof-1865472-supplementary.pdf]

Table S1. *In vitro* papers using antifungal combinations.

Reference	Isolates and Species[a]	Combinations	Methods	Results[b]
**Caballero et al., 2020 [16]**	6 *C. auris*	ISV+ANI or CAS or MICA	CK, TK	100% SYN
**Reginatto et al., 2020 [27]**	3 *C. albicans*, 3 *C. parapsilosis*, 3 *C. tropicalis*	ANI+AMB	CK, Biofilm evaluations	100% SYN
**O'Brien et al., 2020 [33]**	15 *C. auris**	5-FC+AMB or ANI or CAS or POS or ISV or ITZ or VRC or MICA	CK	100% inhibition in 9/15 isolates AMB+5-FC; 6/15 isolates 5-FC+ANI; 6/15 isolates 5-FC+CAS; 6/15 isolates 5-FC+MICA; 13/15 isolates VRC+5-FC; other combinations were indifferent.
**Kovács et al., 2019 [37]**	6 *C. albicans*, 5 *C. parapsilosis*. 1 *C. albicans* and 1 *C. parapsilosis* for biofilm assay*	NIK+MICA or CAS	CK, Biofilm viability assay	CK: NIK+CAS *i*) *C. albicans* 67% SYN, 33% IND. ii) *C. parapsilosis* 100% IND. NIK+MICA: i) *C. albicans* 100% SYN, ii) *C. parapsilosis* 80% SYN and 20% IND. LIVE/DEAD staining: the combinations showed an increase in dead cells.
**Chassot et al., 2019 [31]**	60 *C. parapsilosis**	AMB+5-FC or FLU or VRC, FLU + 5F-C or VRC, VRC + 5-FC	CK	AMB+5FC: 20% SYN, 8% ADD, 50% IND and 22% ANT. AMB+FLU: 5% SYN, 17% ADD, 45% IND and 33% ANT. AMB+VRC: 13% ADD, 45% IND and 42% ANT. FLU+5FC: 7% SYN, 8% ADD, 58% IND and 27% ANT. FLU+VRC: 25% SYN, 23% ADD, 43% IND and 8% ANT. VRC+5FC: 17% SYN, 8% ADD, 53% IND and 21% ANT.
**Cheung et al., 2017 [36]**	4 *C. albicans**	NIK+ANI or MICA	CK	100% SYN
**Denardi et al., 2017 [21]**	10 *C. glabrata**	CAS or MICA or ANI+FLU or AMB or POS or VRC	CK	CAS+FLU: 85% IND and 15% ANT. MICA+FLU: 30% SYN, 55% IND, 15% ANT. ANI+FLU: 45% SYN, 45% IND, 10% ANT. CAS+POS: 85% SYN, 15% IND. MICA+POS: 30% SYN, 70% IND. ANI+POS: 70% SYN, 30% IND. CAS+VRC: 35% SYN, 65% IND. MICA+VRC: 15% SYN, 60% IND, 25% ANT. AMB+VRC: 70% SYN, 30% IND. CAS+AMB: 20% SYN, 70% IND, 10% ANT. MICA+AMB: 15% SYN, 35% IND, 50% ANT. ANI+AMB: 35% SYN, 55% IND, 10% ANT. ANI+VRC: 70% SYN, 30% IND.
**Alvarez et al., 2017 [48]**	3 *C. albicans**	AMB+5-FC	CK, TK	CK: 100% IND. Time kill curves: 100% SYN
**Katragkou et al., 2017 [17]**	4 *C. albicans*, 3 *C. parapsilosis*, 4 *C. glabrata*, 4 *C. tropicalis* and 3 *C. krusei**	ISV+AMB or MICA	CK, TK	ISV+MICA - Bliss assay: 56% SYN, 44% IND. Time kill: 39% SYN, 61% IND. ISV+AMB - Bliss assay: 61 % IND, 17 % SYN and 22% ANT. Time kill: 44.% IND, 17% SYN and 39 % ANT
**Siopi et al., 2015 [32]**	1 *C. parapsilosis*	VRC+CAS or ANI or MICA	DD	100% IND
**Siopi et al., 2015 [15]**	2 *C. albicans*, 2 *C. glabrata*, 2 *C. kefyr*, 2 *C. tropicalis*, 2 *C.*	VRC+CAS or AMB	MTS and CK	MTS: AMB+VRC 95% ANT, 5% SYN. VRC+CAS: 61% ANT, 28% ADD, 11% SYN. CK:

	krusei and 2 *C. parapsilosis*			AMB+VRC 33% SYN, 67% ANT. VRC+ CAS 11% SYN, 89% ADD
**Khodavandi et al., 2014 [40]**	6 *C. albicans**	FLU+TER	CK, TK	FIC 50: 50% SYN, 50% ADD; FIC 90: 50% SYN, 50% ADD
**Steier et al., 2013 [49]**	5 *C. glabrata**	FLU+5-FC	CK	100% ANT
**Kaneko et al., 2013 [39]**	1 *C. albicans*	FLU+BUT or TER or CLI or SER or KTZ or ITZ or MIZ or OXI or TIO	CK	Enhanced: FLU+ BUT or TER. FLU+CLI or SER or KTZ or ITZ or MIZ or OXI or TIO equivalent to or greater than FLU.
**Chen et al., 2013 [19]**	10 *C. albicans**	POS+CAS	CK	100% SYN
**Alves et al., 2012 [22]**	68 *C. glabrata**	AMB+VRC, AMB+CAS, AMB+5-FC, 5-FC+ITZ, 5-FC+VRC, 5-FC+CAS, 5-FC+KTZ, VRC+ITZ and VRC+CAS	CK	CAS+AMB: 26% SYN, 66% IND, 7% ANT. 5-FC+AMB: 69% SYN, 31% IND. VRC+AMB: 31% SYN, 54% IND, 15% ANT. CAS+5-FC: 38% SYN, 60% IND, 1% ANT. KTZ+5-FC: 3% SYN, 38% IND, 59% ANT. ITZ+5-FC: 32% SYN, 54% IND, 13% ANT. VRC+5-FC: 37% SYN, 54% IND, 9% ANT. VRC+ITZ: 28% SYN, 60% IND, 12% ANT. VRC+CAS: 15% SYN, 78% IND, 7% ANT
**Tobudic et al., 2010 [24]**	10 *C. albicans**	AMB+POS; AMB+CAS	CK	AMB+CAS: 40% SYN, 60% IND. AMB+POS: 100% IND
**Pai, 2009 [42]**	2 *C. albicans**	MICA+CAS+AMB, MICA+CAS or AMB, CAS+AMB	TK	Triple combination more effective than double therapy, followed by AMB+MICA.
**Baltch et al., 2008 [50]**	3 *C. glabrata**	VRC+CAS	TK	Combination more effective than single molecules
**Pai et al., 2008 [41]**	1 *C. albicans*, 1 *C. glabrata*, 1 *C. parapsilosis*, and 1 *C. tropicalis**	5-FC+VRC+MICA	TK	Triple combination not superior compared to double combination
**Vakil et al., 2008 [51]**	2 *C. albicans*	AMB+5-FC	CK	100% IND
**Serena et al., 2008 [25]**	35 *C. krusei*, 35 *C. albicans*, 15 *C. parapsilosis*, 15 *C. tropicalis*, 20 *C. dulbiniensis*, 15 *C. glabrata* and 10 *C. lusitaniae*. TK 2 *C. albicans*, 2 *C. glabrata*, 2 *C. parapsilosis**	AMB+MICA	CK, TK	CK MIC-0 end point: *C. krusei* 26% SYN 74% IND. *C. albicans* 8.5% SYN and 91.5% IND. *C. parapsilosis* 40% SYN and 60% IND. *C. tropicalis* 47% SYN and 53% IND. *C. dulbiniensis* 35% SYN and 65% IND *C. glabrata* and *C. lusitaniae* 100% IND. CK MIC-2 end point: *C. krusei* 37% SYN 63% IND. *C. albicans* 71% SYN and 29% IND. *C. parapsilosis* 60% SYN and 40% IND. *C. tropicalis* 53% SYN and 47% IND. *C. dulbiniensis* 50% SYN and 50% IND *C. glabrata* 53% SYN and 47% IND. *C. lusitaniae* 20% SYN and 80% IND. TK: 50% SYN and 50% IND
**Chaturvedi et al., 2008 [52]**	1 *C. kruseii*	AMB+CAS+POS+VRC	CK	100% IND

**Barchiesi et al., 2007 [28]**	3 *C. parapsilosis*	CAS+AMB	DD, TK	100% IND
**Shuford et al., 2007 [53]**	30 *C. albicans*	CAS+VRC	XTT assay	100% IND
**Karlowsky et al., 2006 [14]**	4 *C. albicans*, 4 *C. glabrata*, 4 *C. parapsilosis*, 4 *C. tropicalis* and 2 *C. krusei* *	ANI+FLU or ITZ or KTZ or AMB or 5-FC	CK	ANI+ITZ: 5% SYN and 95% IND. ANI+KTZ: 22% ANT and 78% IND. ANI+AMB 100% ADD. ANI+FLU: 100% IND
**Heyn et al., 2005 [13]**	55 *C. albicans*, 19 *C. dubliniensis*, 12 *C. glabrata* and 12 *C. parapsilosis**	VRC+MICA	CK	*C. albicans*: 2% SYN, 98% IND; *C. dubliniensis* 100% IND; *C. glabrata* 17% SYN, 83% IND; *C. parapsilosis* 100% IND
**Barchiesi et al., 2005 [20]**	2 *C. glabrata**	CAS+AMB	CK	100% IND
**Gil-Lamaignere et al., 2004 [38]**	59 *C. albicans*, 41 *C. dulbiniensis* and 26 *C. kefyr**	CAS+TER	CK	*C. albicans* 67% SYN, 28% ADD and 5% IND; *C. kefyr* 100% SYN and *C. dubliniensis* 100 % IND
**Girmenia et al., 2003 [54]**	1 *C. krusei* and *1 C. glabrata**	FLU+5-FC	TK	100% IND
**Hossain et al., 2003 [26]**	1 *C. albicans**	CAS+AMB	CK	100% IND
**Te Dorsthorst et al., 2002 [34]**	9 *C. albicans*, 9 *C. glabrata*, 9 *C. krusei**	5-FC+ AMB or FLU	CK	5-FC+FLU: *C. albicans* 50% SYN and 50% ANT; *C. krusei* 11% SYN, 89% ANT; *C. glabrata* 100% ANT. 5-FC+AMB: *C. albicans* 44.4% SYN, 56% ANT; *C. krusei* 77% SYN, 22% ANT; *C. glabrata* 33% SYN and 67% ANT
**Roling et al., 2002 [55]**	2 *C. albicans*, 2 *C. krusei*, 2 *C. tropicalis**	FLU+ANI or CAS	TK	100% IND
**Lewis et al., 2002 [29]**	3 *C. albicans*, 1 *C. glabrata*, 1 *C. kruseii*, 1 *C. tropicalis**	AMB+ FLU or 5-FC, FLU+5-FC	CK, TK, E-test	CK: AMB+FLU 17% SYN, 83% IND; AMB+5-FC: 50% SYN, 50% IND; FLU+5-FC: 33% SYN, 67% IND. E-test: AMB+FLU 50% antagonist, 50% IND. AMB+5-FC and FLU+5-FC 100% IND. Time kill curves: 100% IND
**Louie et al., 2001 [30]**	4 *C. albicans*	FLU+AMB	TK	100% ANT

* The study used isolates resistant to at least one antifungal drugs

[b] Interactions for checkerboards were defined as synergistic (SYN) if the FIC index (FICI) was ≤0.5, additive (ADD) if 0.5 < FICI < 1.0, indifferent (IND) if 1.0 ≤ FICI ≤ 4.0, and antagonistic (ANT) if FICI was >4.0. FIC 50 was calculated on 50% of inhibition, FIC 90 was calculated on 90% of inhibition. Interactions for time kill assays were defined as synergistic (SYN) if combination resulted in a CFU reduction >2Log compared to most active drug, indifferent (IND) if the combination yielded a CFU number ≤±2Log compared to the most active drug, and antagonistic if the CFU number of the combinations was higher than 2Log compared to the most active drug. Abbreviations: ISV, isavuconazole; ANI, anidulafungin; CAS, caspofungin; MICA, micafungin; AMB, amphotericin; 5-FC, 5-flucytosine; POS, posaconazole; ITZ, itaconazole; VRC, voriconazole; NIK, nikkomicin Z; FLU, fluconazole; TER, terbinafine; BUT, butenafine; CLI, climbazole; SER, sertaconazole; KTZ, ketoconazole; MIZ, miconazole, OXI, oxiconazole; TIO, tioconazole; CK, Checkerboard titration (performed in broth or otherwise specified); TK, time kill curves; DD, disk diffusion; MTS, MIC test strip.

Table S2. Case reports and clinical trials using antifungal combinations.

Reference	Clinical case	Combinations	Outcome
**Guo et al., 2021 [56]**	4 cases of *C. parapsilosis* prosthetic valve endocarditis	CAS+VRC, CAS+VRC+AMB, VRC+FLU, FLU+CAS	2 patients survived, 2 patients died
**Noguchi et al., 2019 [57]**	A 73-year-old woman with *C. parapsilosis* melanonychia.	ITZ+EFI	Unsuccessful
**Kubota et al., 2018 [59]**	A 31-year-old woman with repaired tetralogy of Fallot and infective endocarditis due to *C. albicans*	MICA+FLU	Full recovery
**Tu et al., 2017 [60]**	*C. glabrata* keratitis after Descemet Membrane Endothelial Keratoplasty	AMB+FLU	Full recovery
**Al-Sweih et al., 2017 [61]**	A 26-week-preterm man with bloodstream infection by *C. conglobata*	AMB+CAS	Full recovery
**Carrega et al., 2017 [62]**	A 68-year-old diabetic woman with hip arthroplasty with *C. albicans* recovered from a fistula swab.	AMB+ANI	Treatment discontinued due to side effects
**Scemla et al., 2016 [63]**	A 28-year-old man patient with aortic endocarditis and periaortic abscess due to *C. parapsilosis.*	AMB+5-FC	Full recovery
**Charlier et al., 2015 [64]**	Case 1. A 66-year-old woman presented with symptomatic *C. glabrata* cystitis. Case 2. A 65-year-old man presented with *C. glabrata* cystitis.	CAS+5-FC	Microbiological failure
**Herbst et al., 2015 [65]**	A 4-year-old with *C. albicans* meningitis	AMB+5-FC+VRC	Microbiological recovery
**Garcia et al., 2015 [66]**	A 39-year-old man with candiduria from *C. parapsilosis*	CAS+5-FC	Full recovery
**Valentine et al., 2014 [67]**	An 8-week-old male infant with disseminated *C. lusitaniae* septic shock and multisystem organ failure	AMB+5-FC+FLU	Full recovery
**DiMondi et al., 2014 [68]**	a 64-year-old woman in hemodialysis with *C. albicans* blood stream infection.	AMB+MICA then AMB+FLU	Full recovery
**Ruiz-Ramos et al., 2014 [69]**	A 34-year-old woman with chronic femoral osteomyelitis and persistent suppuration, developed a *C. albicans* infection, isolated in the fistula exudate cultures	FLU+ANI then MICA+FLU	Full recovery
**Hagiya et al., 2013 [70]**	An 85-year-old woman presenting with right internal jugular vein *C. albicans* thrombophlebitis catheter-associated.	MICA+ITZ switch MICA+FLU	Full recovery
**Jarque et al., 2013 [71]**	6 cases of invasive *C. krusei* candidiasis	CAS+VRC; CAS+AMB	Full recovery
**Lefort et al., 2012 [45]**	*Candida* endocarditis: 11 *C. albicans*, 9 *C. parapsilosis*, 3 *C. tropicalis*, 1 *C. orthopsilosis*, 2 *C. guillermondii*, 2 *C. glabrata*, 1 *C. kefyr and* 1 *C. pelliculosa*	FLU+5-FC, FLU+AMB, FLU+CAS, CAS+AMB, CAS+5-FC, CAS+VRC, AMB+5-FC	16.7% clinical success. 83. 3% death or no therapeutic success.
**Chan et al., 2012 [72]**	Infectious endophthalmitis: 1 *C. parapsilosis* and 1 *C. albicans*	AMB+FLU	1 patient did not recover, 1 patient improved.

**Cheng et al., 2011 [73]**	A 4-day-old female infant with blood stream infection and peritonitis by *C. parapsilosis*	AMB+FLU+5-FC+CAS	Full recovery
**Radike et al., 2011 [74]**	A 10-month-old girl suffering from cystic fibrosis and osteoarticular infection by *C. albicans*	5-FC+FLU	Full recovery
**Kumar et al., 2011 [75]**	*C. parapsilosis* endocarditis in a 54-year-old man with a history of HIV and Hepatitis C infection	AMB+5-FC	Clinical failure and valve replacement
**Mahdy et al., 2010 [76]**	Fungal keratitis by 7 *C. albicans*	AMB+FLU	Microbiological and clinical recovery
**Mahdy et al., 2010 [77]**	Fungal keratitis by 5 *C. albicans*	AMB+FLU	Microbiological and clinical recovery
**Okamoto et al., 2010 [78]**	A 22-month-old boy with blood culture positive for *C. tropicalis*.	VRC+MICA	Full recovery
**Chew et al., 2010 [79]**	A 72-year-old woman post-operative *C. parapsilosis* endophthalmitis.	AMB+VRC	Full recovery
**Glick et al., 2010 [80]**	A 33-year-old woman with cerebrospinal fluid cultures positive for *C. albicans* and methicillin-resistant coagulase-negative *Staphylococcus spp*.	AMB+5-FC	Microbiological and clinical recovery
**Bernbeck et al., 2009 [81]**	A 15-year-old boy with fulminant *C. krusei* sepsis complicated by acute blindness due to enophthalmitis and subsequent bleeding during prolonged pancytopenia after induction therapy.	AMB+5-FC	Full recovery
**Haase et al., 2009 [82]**	Preterm infant with severe congenital ichthyosis and sepsis caused by *C. albicans*.	AMB+CAS	Full recovery
**Varisco et al., 2009 [83]**	An 11-day-old female infant with Hirschsprung enterocolitis and bowel perforation. *C. albicans* grew from cultures obtained on abdominal washout	AMB+ANI	Full recovery
**Falcone et al., 2009 [84]**	*Candida* infective endocarditis: 3 *C. parapsilosis*, 1 *C. albicans*, 1 *C. famata*	CAS+FLU+POS; CAS+FLU; CAS+AMB; CAS+ITZ; CAS+VRC	3 deaths, 2 recovery
**Bland et al., 2009 [85]**	A 55-year-old woman with total left knee arthroplasty and knee infection by *C. albicans*	MICA+FLU	Relapse and prothesis substitution after 8 weeks
**Wellinghausen et al., 2009 [86]**	A 19-year-old man with congenital haemolytic anaemia and peripheral blood stem cell transplantation. Generalized papulo-pustulous skin efflorescences, suggestive of septic metastases by *C. dubliniensis*.	AMB+CAS	Clinical failure
**Albano et al., 2009 [87]**	3 transplanted patients with *C. albicans* infection	FLU+CAS, FLU+CAS+AMB, FLU+AMB	Full recovery
**Wong et al., 2008 [46]**	13 patients with fungal peritonitis: 2 *C. albicans*, 8 *C. parapsilosis* and 3 *C. glabrata*	AMB+5-FC	6 deaths
**Karatza et al., 2008 [88]**	Two premature infants with invasive candidiasis and endocarditis by *C. albicans*	AMB+FLU	Microbiological and clinical recovery
**Gahn et al., 2007 [89]**	26-years-old man with leuakemia and systemic candidiasis by *C. krusei*	AMB+VRC+CAS	Microbiological and clinical recovery
**Kanavi et al., 2007 [90]**	21-year-old man with keratitis by *C. glabrata* after keratoplasty	AMB+KTZ	Full recovery
**Olver et al., 2006 [91]**	A 24-year-old man leukemic patient with *C. krusei* fungemia	CAS+AMB	Microbiological and clinical recovery

**Paula et al., 2006 [92]**	2 newborns patients recovered in neonatal ICU with Pichia anomala fungemia	AMB+FLU, AMB+ 5-FC	2 deaths
**Al-Assiri et al., 2006 [93]**	A 69-year-old man with keratitis by *C. glabrata* after keratoplasty	AMB+MICA+FLU	Clinical recovery
**Pelletier et al., 2005 [94]**	A 49-year-old woman with intraoperative culture of abdominal fluid positive for *C. krusei*	AMB+5-FC	Clinical recovery
**Lye et al., 2005 [95]**	A 72-year-old man with *C. glabrata* prosthetic mitral valve endocarditis	FLU+CAS	Microbiological recovery
**Fourtounas et al., 2006 [96]**	A 65-year-old man with urinary-tract catheter infection by *C. albicans*	AMB+CAS	Microbiological recovery
**Ostrosky-Zeichner et al., 2005 [44]**	Adult patiens with diagnosis of candidemia: *C. albicans, C. glabrata, C. parapsilosis, C. tropicalis, C. krusei*	MICA+Azole or AMB or both	Microbiological and clinical recovery: 71.4 - 85.7%
**Wagner et al., 2005 [97]**	A 66-years-old man with immunosuppressive therapy and bronchoalveolar lavage positive for *Debaryomyces hansenii*	AMB+CAS	Death
**Natarajan et al., 2005 [43]**	13 infants with blood cultures positive for *Candida*: 5 *C. albicans*, 6 *C. parapsilosi*, 1 *C. tropicalis and 1 C. albicans*	CAS+AMB or FLU or 5-FC	Microbiological recovery: 11/13 patients
**Breit et al., 2005 [98]**	Case 1: A 66-year-old woman with adenocarcinoma of the colon *C. glabrata* septicemia. Case 2: A 42-year-old IV man drug abuser with a history of *C. albicans* septicemia. Patient 3: A 48-year-old woman with ovarian and lung cancer, chronic malnutrition, and C. *albicans* septicemia	VRC+CAS	Full recovery
**Solomon et al., 2004 [99]**	A 51-year-old white woman with *C. parapsilosis* following laser in situ keratomileusis	AMB+FLU	Full recovery
**Muallem et al., 2003 [100]**	A 64-year-old man with bilateral *C. parapsilosis* interface keratitis after laser in situ keratomileusis	AMB+5FC	Full recovery
**Mikamo et al., 2003 [101]**	29-year-old woman, neutropenic and with tuboovarian abscess caused by *C. glabrata* after chemotherapy	FLU+AMB	Full recovery
**Girmenia et al., 2003 [54]**	Case 1: A 46-year-old man with acute myelogenous leukemia and isolation of *C. glabrata* in CVC and blood. Case 2: A 10-year-old child with severe aplastic anemia, bone marrow transplantation and hemorrhagic cystitis by *C. glabrata**	FLU+5-FC	Full recovery
**Shann et al., 2003 [102]**	28-year-old woman with a 10-year history of recurrent *candida* with candida vaginosis by *C. glabrata*	AMB+5-FC	Full recovery
**Rex et al., 2003 [47]**	Patients >13 years old with candidemia: 68 *C. albicans*, 18 *C. glabrata*, 16 *C. parapsilosis*, 13 *C. tropicalis*, 2 *C. lusitaniae* and 1 *C. kefyr*.	FLU+AMB	Clinical failures: *C. albicans* 34%, *C. glabrata* 50%, *C. parapsilosis* 19%, *C. tropicalis* 23%, *C. lunsitaniae* 50%
**Sutphin et al., 2002 [103]**	15-years-old man with *C. albicans* Keratitis after keratoplasty	AMB+FLU	Microbiological recovery
**Ramamohan et al., 2001 [104]**	A 65-year-old woman with hip infection by *C. glabrata*.	AMB+5-FC	Full recovery

**Soto-Hernández et al., 2000 [105]**	A 22-year-old woman with ventriculoatrial cerebrospinal fluid shunt placement and *C. albicans*	AMB+KTZ	Full recovery

Abbreviations: CAS, caspofungin; AMB, amphotericin B; VRC, voriconazole; FLU, fluconazole; ITZ, itraconazole; EFI, eficonazole; MICA, micafungin; 5-FC, 5-flucytosine; ANI, anidulafungin; KTZ, ketoconazole; ICU, intensive care unit.
